# *β*-Catenin is involved in alterations in mitochondrial activity in non-transformed intestinal epithelial and colon cancer cells

**DOI:** 10.1038/sj.bjc.6605342

**Published:** 2009-10-13

**Authors:** M Mezhybovska, Y Yudina, A Abhyankar, A Sjölander

**Affiliations:** 1 Cell and Experimental Pathology, Department of Laboratory Medicine, Malmö University Hospital, Lund University, Malmö, SE-205 02, Sweden; 2 Department of Clinical Sciences, Clinical Research Centre, Malmö University Hospital, Lund University, Malmö, SE-205 02, Sweden

**Keywords:** β-catenin, mitochondrial activity, mtDNA transcription, epithelial cells, colon cancer, leukotriene D4

## Abstract

**Background:**

Alteration in respiratory activity and mitochondrial DNA (mtDNA) transcription seems to be an important feature of cancer cells. Leukotriene D_4_ (LTD_4_) is a proinflammatory mediator implicated in the pathology of chronic inflammation and cancer. We have shown earlier that LTD_4_ causes translocation of *β*-catenin both to the mitochondria, in which it associates with the survival protein Bcl-2 identifying a novel role for *β*-catenin in cell survival, and to the nucleus in which it activates the TCF/LEF transcription machinery.

**Methods:**

Here we have used non-transformed intestinal epithelial Int 407 cells and Caco-2 colon cancer cells, transfected or not with wild type and mutated (S33Y) *β*-catenin to analyse its effect on mitochondria activity. We have measured the ATP/ADP ratio, and transcription of the mtDNA genes ND2, ND6 and 16 s in these cells stimulated or not with LTD_4_.

**Results:**

We have shown for the first time that LTD_4_ triggers a cellular increase in NADPH dehydrogenase activity and ATP/ADP ratio. In addition, LTD_4_ significantly increased the transcription of mtDNA genes. Overexpression of wild-type *β*-catenin or a constitutively active *β*-catenin mutant mimicked the effect of LTD_4_ on ATP/ADP ratio and mtDNA transcription. These elevations in mitochondrial activity resulted in increased reactive oxygen species levels and subsequent activations of the p65 subunit of NF-*κ*B.

**Conclusions:**

The present novel data show that LTD_4_, presumably through *β*-catenin accumulation in the mitochondria, affects mitochondrial activity, lending further credence to the idea that inflammatory signalling pathways are intrinsically linked with potential oncogenic signals.

Leukotriene D_4_ (LTD_4_) is a powerful proinflammatory mediator derived from arachidonic acid through the 5-lipoxygenase pathway. Arachidonic acid is the common precursor of a group of mediators collectively called eicosanoids (Funk, 2001). Leukotriene D_4_ is the most potent of the cysteinyl leukotrienes (LTC_4_, LTD_4_, and LTE_4_). Leukotriene D_4_ is known to mediate its effects through specific cell surface receptors belonging to the G protein-coupled receptor family. The CysLT_1_ and CysLT_2_ receptors has been most studied (Heise *et al*, 2000; Lynch *et al*, 1999; Sarau *et al*, 1999) of which, the CysLT_1_ receptor has been proven to have a much higher affinity for LTD_4_ (Heise *et al*, 2000). Leukotriene D_4_ signalling has been implicated in chronic inflammatory conditions such as asthma and inflammatory bowel diseases (IBDs) (Drazen, 2002).

Inflammatory bowel diseases are associated with an increased incidence of neoplastic transformation (Ekbom *et al*, 1990), suggesting that there is a link between inflammation and cancer (Sheng *et al*, 1997). Furthermore, earlier studies have shown that colon cancer is underrepresented in a population of patients with ulcerative colitis, who where treated with non-steroidal anti-inflammatory drugs (Smalley and DuBois, 1997). This suggests that inflammatory mediators, such as LTD_4,_ could be essential factors in mediating the coupling between IBD and colon cancer.

In 1930, Warburg showed that some types of cancer cells are capable of constitutive upregulation of glucose metabolism, even in the presence of the abundant oxygen. This phenomenon is known as the Warburg effect (Warburg, 1956). Cancer cells synthesise ATP mainly through ‘anaerobic glycolysis’, a metabolic state that is linked to high glucose uptake and local acidification due to lactate production, even in the presence of oxygen. Cancer cells often upregulate glycolysis enzymes as a result of constitutive signalling through the Akt pathway, or because of the expression of oncogenes such as Ras or Src (Elstrom *et al*, 2004; Pelicano *et al*, 2006). Earlier we have shown that LTD_4_ induces Akt phosphorylation in Int 407 cells (Paruchuri *et al*, 2005).

A major finding in understanding increases in respiration is based on the well-established fact that there is an increase in the average of ATP/ADP ratio (Matsunaga *et al*, 1996). The stimulation of both respiration (oxidative phosphorylation) and glycolysis is presumably responsible for the rise in the ATP/ADP ratio, which begins almost simultaneously with the increase in respiration.

Certain types of chronic inflammation such as ulcerative colitis have long been associated with a high risk of cancer development (Coussens and Werb, 2002; Hussain *et al*, 2003). It is believed that increased free radical generation is one important mechanism that promotes the progression of chronic inflammation into malignant transformation (Hussain *et al*, 2003). Cancer patients commonly have decreased glucose clearance capacity, high glycolytic activity and raised lactate production. Therefore, it has been suggested that the observed pro-oxidative shift is mediated by an increased availability of mitochondrial energy substrate. The ‘inflammatory oxidative conditions’ are typically associated with an excessive stimulation of NAD(P)H oxidase by cytokines and other inflammatory mediators (Kohli *et al*, 2007). Increased reactive oxygen species (ROS) production or changes in intracellular glutathione levels are often involved with pathological changes, both of which are indicative of signal cascade, or gene expression dysregulation (Waris and Ahsan, 2006).

*β*-Catenin is a multifunctional protein, its function is altered in most colon cancers. In non-transformed cells it is present at the cell membrane, where together with E-cadherin forms part of the adherent-type junction (Conacci-Sorrell *et al*, 2002; Cullen *et al*, 2004). In the presence of a Wnt signal, *β*-catenin translocates to the nucleus in which it activates transcription factors of the TCF/LEF family (Korinek *et al*, 1997). *β*-Catenin has an essential role in normal cell physiology, which makes it an unsuitable target for antagonising with specific inhibitors or siRNA. We have shown earlier that *β*-catenin translocates to the nuclei of Int 407 cells after exposure to the pro-inflammatory mediator LTD_4_ (Mezhybovska *et al*, 2006). We also found that after LTD_4_ stimulation, *β*-catenin was present in mitochondria where it was associated with the anti-apoptotic protein, Bcl-2 (Mezhybovska *et al*, 2006). Here we further examine the effects of *β*-catenin on mitochondrial activity.

## Materials and methods

### Constructs

HA-wt-*β*-catenin (*β*-cat wt, wild type) and HA-S33Y-*β*-catenin (*β*-cat S33Y, constitutively active mutant) constructs were generously provided by Dr Ben-Zeev (Weizmann Institute of Science, Rehovot, Israel). The NF*κ*B-RE luciferase construct was from Promega (Madison, WI, USA).

### Cell culture

The non-transformed human intestinal epithelial cell line (Int 407) which shows typical epithelial morphology and growth, was isolated from jejunum and ileum of a human embryo of 2 months gestation (Henle and Deinhardt, 1957). Int 407 cells were cultured as a monolayer in Eagle's basal medium supplemented with 15% newborn calf serum. The colon cancer cell line Caco-2 was grown in Dulbecco's modified Eagle medium (Sigma Chemicals Co, St Louis, MO, USA) with 10% fetal bovine serum (Wikström *et al*, 2003). All media was supplemented with 2 mM L-glutamine, 55 IU ml^–1^ penicillin and 55 *μ*g ml^–1^ streptomycin. The cell lines were cultured at 37°C in a humidified atmosphere with 5% CO_2_. The cells were regularly tested to ensure the absence of mycoplasma contamination.

### Transfection

Transfection was performed in complete medium for 24 h using PolyFect transfection reagent (Qiagen GmbH, Hilden, Germany), according to the manufacturer's instructions. In all transfection experiments it was routinely confirmed that empty vector had no effect.

### Reverse transcription–PCR

Messenger RNA (mRNA) for the p65 NF-*κ*B subunit was synthesised using the forward primer: GCGAGAGGAGCACAGATACCACCAA and the reverse primer: GGCAGATCTTGAGCTCGGCAGTGTT.

### mRNA isolation cDNA synthesis and quantitative real-time PCR

Messenger RNA was isolated using the RNeasy Plus Mini Kit from Qiagen following the manufacturer's instructions. Complementary DNA (cDNA) was synthesised using the Fermentas Revert Aid H Minus First Strand cDNA Synthesis kit following the manufacturer's instructions, or with random hexamer primers and superscript II reverse transcriptase (Invitrogen, Carlsbad, CA, USA), according to the standard procedures. Complementary DNA was used as a template in quantitative real-time PCR reactions with TaqMan Gene Expression Assay (Applied Biosystems, Cambridge, UK) and Maxima Probe qPCR Mastermix (Fermentas Life Sciences, Vilnius, Lithuania). For relative quantification of expression levels, the comparative *C*_t_ method was used. The point at which the fluorescence crosses the threshold was taken as the [*C*_t_*]* value. Expression levels of the different genes of interest were normalised to the expression level of the housekeeping gene, *β*-actin or GAPDH.

### NADPH activity determination (MTS assay)

Cells were grown to 60% confluency. They were then stimulated or not with 40 nM LTD_4_ for the indicated period of times. Assays were performed using the Cell Titer 96 assay kit from Promega, following the manufacture's instructions. Conversion of MTS (3-(4,5-dimethylthiazol-2-yl)-5-(3-carboxymethoxyphenyl)-2-(4-sulfophenyl)-2H-tetrazolium) into aqueous, soluble formazan was measured by recording absorbance at 490 nm using a 96-well plate reader.

### ATP/ADP ratio determination

The transfected and untransfected cells were grown to 60% confluency, and thereafter stimulated or not with 40 nM LTD_4_. Cells were collected in 20% TCA and frozen on dry ice for ATP determination. ATP was determined using the ATP Kit SL (luminescent assays) from BioThema (Handen, Sweden). Untreated samples were diluted 40-fold and measured bioluminometrically. Thereafter, ATP was irreversibly converted to AMP with ATP sulfurylase in the presence of molybdate. In all, 20 *μ*l of sample were mixed with 180 *μ*l of Buffer I (50 mM/5 mM Tris-HCl/MgCl_2_, pH 8.0, 10 mM Na_2_MoO_4_, 2.5 mM GMP, and 0.5 U ATP sulfurylase), incubated for 20 min at 30°C, boiled for 2.5 min and chilled on ice. Samples were diluted with 800 *μ*l milliQ-water and ATP was measured bioluminometrically. ADP was converted to ATP by pyruvate kinase. In total, 100 *μ*l of the diluted sample from the previous step was mixed with 100 *μ*l of the Buffer II (50 mM/5 mM Tris-HCl/MgCl_2_, pH 8.0, 38 mM KCl, 0.5 mM phosphopyruvate, and 50 U pyruvate kinase), samples were incubated at room temperature for 30 min. To terminate the reaction 300 *μ*l of H_2_O was added and ATP was measured bioluminometricaly (Lundin, 2000). ADP was calculated as the difference between the ATP measured after incubation with pyruvate kinase, and the residual ATP measured after treatment with sulfurylase alone.

### Transient transfection and luciferase assays

Luciferase assays were carried out using the Dual Luciferase Reporter Assay System from Promega. Each plasmid was used at a final concentration of 1 *μ*g ml^–1^, except for the control *Renilla* luciferase vector, which was always used at 0.2 *μ*g ml^–1^ to standardise for transfection efficiency. Vector DNA was allowed to form complexes with PolyFect. Cells in 12-well plates were washed once in serum-free medium, and the DNA-Polyfect mixture was added. Cells were transfected at 37°C for 24 h in complete medium, after which the medium was changed to normal growth medium, and the cells allowed to recover for 24 h. Before any further stimulation, the cells were left in serum-free medium for 1 h. The cells were then pre-treated or not with 30 *μ*M NAC (N-acetyl-L-cysteine, Sigma Chemicals Co, an antioxidant capable of neutralising ROS) for 15 min and then incubated in the absence or presence of 40 nM LTD_4_ for the indicated period of time. After stimulation with LTD_4_, cells were washed in PBS and lysed using 250 *μ*l well^–1^ of the DLR passive lysis buffer provided in the assay. Lysed samples were collected and briefly centrifuged to precipitate the debris. A 20 *μ*l volume of each lysate was used to measure luciferase activity with 50 *μ*l luciferase assay substrate using a MiniLumat LB 9506 (Berthold Technologies, Hannover, Germany) luminometer. The control *Renilla* luciferase signal was recorded after the subsequent addition of 50 *μ*l of Stop&Glow buffer, and the level of expression given as a ratio. In every experiment, triplicate samples were prepared and analysed for each condition.

### Dihydroethidium staining

The oxidative fluorescent dihydroethidium (DHE) dye was used to evaluate the intracellular production of O_2_^−•^ (Kitada *et al*, 2003). Dihydroethidium is cell permeable and reacts with O_2_^−•^ to form ethidium, which in turn intercalates with DNA, providing nuclear fluorescence at an excitation wavelength of 520 nm and emission wavelength of 610 nm. The cells were grown to 60% confluency, and were transfected with HA-S33Y-•*β*-catenin or HA-wt-•*β*-catenin. After 24 h, the cells were serum starved overnight and the following day incubated with 50 *μ*M DHE at 37°C for 30 min. Then the cells were washed with PBS and serum-free culture media was added before fluorescent microscopy (Kohli *et al*, 2007). Exogenous addition of H_2_O_2_ was used as a positive control. All images shown are of living cells maintained under physiological conditions by fluorescent microscopy using an Olympus microscope IX81 (Hamburg, Germany), objective × 20.

### Western blot

The cells were lysed and scraped loose with ice-cold lysis buffer (50 mM Tris, pH 7.5, 1 mM EDTA, 1 mM EGTA, 1 mM Na_3_VO_4_, 1% Triton X-100, 50 mM NaF, 5 mM sodium pyrophosphate, 10 mM sodium glycerophosphate, 4 *μ*g ml^–1^ leupeptin, and 30 *μ*g ml^–1^ phenylmethanesulfonyl fluoride (PMSF)). The resultant lysate was boiled with sample buffer (62 mM Tris pH 6.8, 1.0% SDS, 10% glycerol, 15 mg ml^–1^ dithiothreitol, and 0.05% bromphenol blue) for 10 min. Equal amounts of protein (30–50 *μ*g protein well^–1^) were loaded and subjected to electrophoresis on 8% homogeneous polyacrylamide gels. The separated proteins were electrophoretically transferred to PVDF membranes, which were then blocked for 1 h at ambient temperature with 3% BSA/PBS. The membranes were then incubated overnight at 4°C with the primary antibody (*β*-catenin diluted 1 : 500). The membranes were washed and incubated for 1 h with HRP-conjugated secondary antibody, diluted 1 : 5000 in 3% BSA/PBS with 0.1% Tween-20. Membranes were incubated with ECL western blot detection reagents, and exposed to Hyperfilm-ECL to visualise immunoreactive proteins (Mezhybovska *et al*, 2006).

### Cell fractionation

Treatments were terminated by the addition of ice-cold lysis buffer (20 mM NaHepes pH 8.0, 2 mM MgCl_2_, 1 mM EDTA, 5 mM orthovandate, 60 *μ*g ml^–1^ PMSF and 4 *μ*g ml^–1^ leupeptin) and the cells were placed on ice. After N_2_-decompression at 1000 psi for 10 min using a cell disruption bomb (Parr Instrument Company, Moline, IL, USA) and centrifugation at 200 **g**, the supernatant was centrifuged at 10 000 **g** for 10 min, and further centrifugation at 200 000 **g** for 1 h, to obtain an isolated cytosolic fraction.

## Results

### Leukotriene D_4_ increases metabolic activity in mitochondria

**Figure 1:**
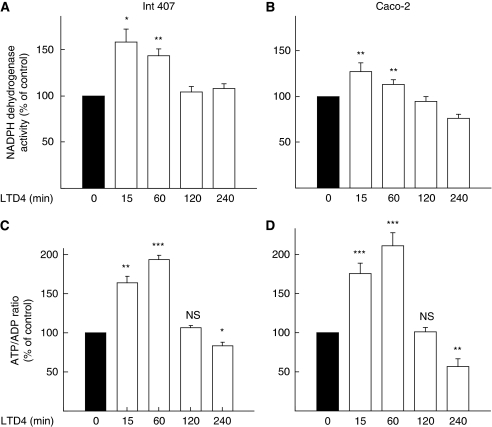
Leukotriene D_4_ (LTD_4_)-induced increase in mitochondrial activity. Int 407 (**A**, **C**) and Caco-2 (**B**, **D**) cells were pre-incubated for indicated periods of time with 40 nM LTD_4_. (**A**, **B**) Cells cultured in complete medium in 96-well plates were subjected to a MTS assay (NADPH dehydrogenase activity) according to the manufacturer's instructions. Conversion of MTS into aqueous soluble formazan was measured by recording the absorbance at 490 nm using a 96-well plate reader. (**C**, **D**) Cells were collected in 20% TCA in PBS, and fast frozen. Thereafter, ATP and ADP amounts were measured as described in the Materials and Methods section. The presented data are given as means±s.e. of three separate experiments. ^*^P<0.05; ^**^P<0.01; ^***^P<0.005.

We have shown earlier that LTD_4_ increases cell survival and causes translocation of *β*-catenin to mitochondria. Therefore, we analysed whether LTD_4_ could affect mitochondrial activity. We observed a significant increase in the NADPH dehydrogenase activity in both the non-transformed epithelial cell line Int 407 and the cancer cell line Caco-2 (Figures 1A and B) in response to the LTD_4_ stimulation (40 nM). The maximum effect was reached after 15 min of stimulation with a significant increase after 1 h. The activity declined again after 2 h of stimulation. As a consequence of increased metabolism in the cell, an increase in the ATP/ADP ratio occurs, which contributes to plasma membrane depolarisation followed by activation of calcium channels leading to an increase in intracellular calcium (Fridlyand *et al*, 2005). Leukotriene D_4_ increased intracellular calcium levels in both Int 407 and Caco-2 cells (Nielsen *et al*, 2005). We also found an increase in the ATP/ADP ratio that was delayed compared with NADPH dehydrogenase activity, with a maximum effect after 1 h. The change was statistically significant at time points between 15 min and 1 h of LTD_4_ stimulation in both cell lines (Figures 1C and D).

### Leukotriene D_4_ increases mitochondrial gene transcription

**Figure 2:**
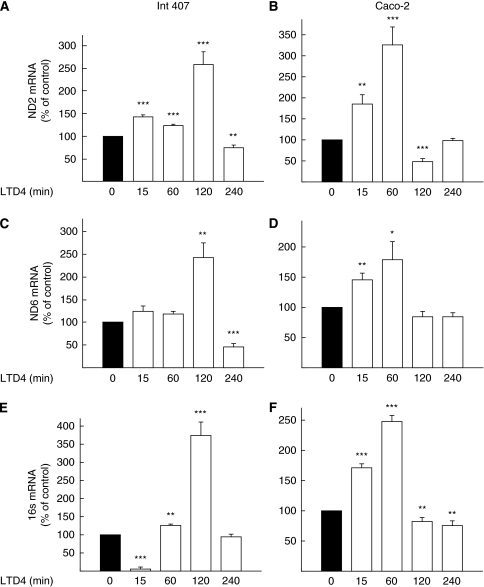
Expression of the mitochondrial-encoded genes upon leukotriene D_4_ (LTD_4_) stimulation. Levels of ND2 (**A**, **B**), ND6 (**C**, **D**), and 16 s (**E**, **F**) messenger RNA (mRNA) in Int 407 (**A**, **C,** and **E**) and Caco-2 (**B**, **D,** and **F**) cells, were measured by real-time PCR after stimulation with 40 nM LTD_4_ for the indicated periods of time. Expression levels of the genes of interest were normalised to the expression of the reference gene *β*-actin. The presented data are given as means±s.e. of three separate experiments. ^*^P<0.05; ^**^P<0.01; ^***^P<0.005.

Next, we examined whether LTD_4_ had any effect on mitochondrial gene transcription. The mitochondrial DNA (mtDNA) is present at a high copy number per cell. It contains genes encoding 13 polypeptides, essential for protein synthesis in mitochondria, and a non-coding region called the displacement loop (D-loop). The D-loop is involved in the control of replication and transcription of mtDNA. The two mtDNA strands differ in their G+T content and can therefore be separated into a heavy strand (H-strand), and a light strand (L-strand). In human cells each strand contains one single promoter for transcriptional initiation (Clayton, 1991; Ojala *et al*, 1981), and an additional initiation site for heavy strand transcription (Montoya *et al*, 1983). We chose to measure mRNA of one gene from each transcript of the two strands of the D-loop that control replication and transcription of mtDNA, in order to detect changes in mitochondrial-encoded gene activity. We observed a significant increase in mRNA for ND2 (NADH dehydrogenase subunit 2) both in Int 407 and Caco-2 cells (Figures 2A and B). This increase in transcription level occurred after 15 min of LTD_4_ stimulation, with the maximum three-fold increase observed after 2 h in Int 407 cells and after 1 h in Caco-2 cells. The same was seen in both cell lines for the other gene transcripts, ND6 (NADH dehydrogenase subunit 6) and 16 s, with the maximum effect after 2 h stimulation with 40 nM LTD_4_ in Int 407 cells and 1 h for the Caco-2 cells (Figures 2C-E and F). The maximum increase (three-fold) was found for 16 s mRNA in Int 407 cells after 2 h of LTD_4_ treatment (Figure 2E).

### *β*-Catenin affects mitochondrial activity

**Figure 3:**
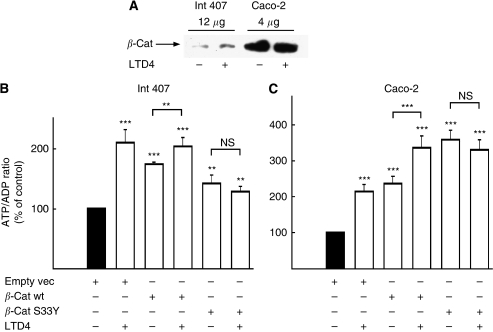
Overexpression of *β*-catenin increases ATP/ADP ratio. (**A**) The endogenous levels of *β*-catenin in Int 407 and Caco-2 cells cytosolic fractions. For the Caco-2 cells only one-third of total protein was added compared with the Int 407 cells, in order to evaluate the amount of *β*-catenin present in the cytosol. (**B**) Levels of overexpressed *β*-catenin HA-wt-*β*-catenin (*β*-cat wt) and HA-S33Y-*β*-catenin (*β*-cat S33Y) mutant in Int 407 cells. Int 407 (**C**) and Caco-2 (**D**) cells were transiently transfected with empty vector (empty vec), *β*-cat wt or *β*-cat S33Y, and treated or not treated with 40 nM LTD_4_ for 1 h. Thereafter, cells were collected in 20% TCA in PBS, and fast frozen. ATP and ADP amounts were measured as described in the Materials and methods section. The presented data are given as means±s.e. of four separate experiments. ^*^P<0.05; ^**^P<0.01; ^***^P<0.005.

Int 407 is a non-transformed cell line showing epithelial cell morphology, in which *β*-catenin is mainly present at the sites of cell–cell junctions (i.e., at the plasma membrane) and follows a normal degradation pattern (Mezhybovska *et al*, 2005). The Caco-2 cell line, however, has a mutation in the APC gene. APC is a part of the *β*-catenin degradation complex. Caco-2 cells have a non-functional *β*-catenin degradation pathway, leading to accumulation of *β*-catenin in the cells. As seen in Figure 3A, the endogenous level of *β*-catenin in the Caco-2 cells is significantly higher than in Int 407 cells (12 *μ*g of total protein from Int 407 cells and 4 *μ*g from Caco-2 cells were loaded on the gel). Next we examined whether *β*-catenin affects mitochondrial activity. We have shown that *β*-catenin translocates to the mitochondria and interacts with the survival protein Bcl-2 (Mezhybovska *et al*, 2006). We overexpressed the following *β*-catenin constructs: *β*-cat wt and *β*-cat S33Y, and measured ATP/ADP ratio in the cells. It is worth mentioning that both *β*-catenin constructs are expressed equally in the cells (data not shown). Overexpression of both wild-type *β*-cat wt and mutant *β*-cat S33Y gave at least a 1.5-fold increase in ATP/ADP ratio in Int 407 cells (Figure 3B) and a 2.5-fold increase in Caco-2 cells (Figure 3C). This effect was almost totally dependent on the *β*-catenin-mediated stimulation of the mitochondrial activity and not on the increase of number of the mitochondria, as seen from Figure 1A. The increase in viable cell number after wt or S33Y *β*-catenin overexpression were <0.2-fold after 24 h (118±11% (wt) and 122±10% (S33Y) of that seen after transfection with empty vector), which is <15% of the total increase in ATP/ADP ratio. Interestingly, LTD_4_ stimulation of cells overexpressing the *β*-cat wt construct caused a further increase in ATP/ADP ratio compared with unstimulated cells. However, LTD_4_ stimulation in cells overexpressing the *β*-cat S33Y construct did not cause any significant changes in mitochondrial activity (Figures 3B and C). The empty vector control had no effect.

The basal expression levels of mitochondrial-encoded genes were different between Int 407 and Caco-2 cells. The basal levels of mitochondrial gene expression were significantly higher in Caco-2 cells (Table 1A). The difference in *β*-catenin levels in the two cell lines obviously correlated with the basal expression profiles of their mitochondrial-encoded genes.

In Int 407 cells, there was a dramatic increase in the expression levels of all three genes ND2, ND6 and 16 s after transfection with *β*-cat wt or *β*-cat S33Y (Table 1B). These stimulated levels in Int 407 cells were compatible to basal expression levels of Caco-2 cells. After the same treatment in Caco-2 cells, the effect of LTD_4_ on gene activity was less prominent. Activity of the ND2 and ND6 genes was not significantly different from cells transfected with empty vector, but 16 s transcription was increased 1.5-fold (Table 1C). In the Int 407 cells that had been transfected with wild-type or mutant *β*-catenin, LTD_4_ stimulation induced a significant increase in ND2, ND6 and 16 s transcription. Stimulation of Caco-2 cells overexpressing wild-type *β*-catenin was not significantly different from the unstimulated cells for ND2, ND6 and 16 s expression. Stimulation of Caco-2 cells that overexpressed constitutively active *β*-catenin, resulted in a significant increase compared with unstimulated cells (the effect was 1.5-fold for all analysed genes). The difference in the response between Int 407 and Caco-2 cells to LTD_4_ stimulation might be explained by the substantial difference in basal levels of gene expression between the two cell lines (Table 1A).

### *β*-Catenin-mediated increase in mitochondria activity leads to NF-*κ*B activation through ROS

**Figure 4:**
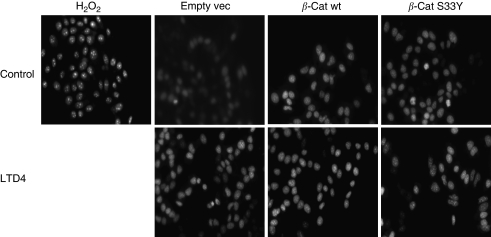
Detection of O_2_^−•^ production by dihydroethidium (DHE) staining in Int 407 cells. Representative results of DHE staining are shown from three independent experiments Int 407 cells (with or without overexpression of HA-wt-*β*-catenin (*β*-cat wt) or HA-S33Y-*β*-catenin) stimulated or not with 40 nM LTD_4_ for 1 h. The mean fluorescent intensity per cell in empty vector transfected cells was 11.2 RU, the mean value per cell for H_2_O_2_ positive control 26.5 RU, and for the LTD_4_ stimulation in Int 407 cells was 16.1 RU, mean values for other treatments were not different from LTD_4_ stimulation. Before imaging, the cells were preincubated with DHE for 30 min. The fluorescent micrographs show living cells under physiological conditions with 20 × objective.

**Figure 5:**
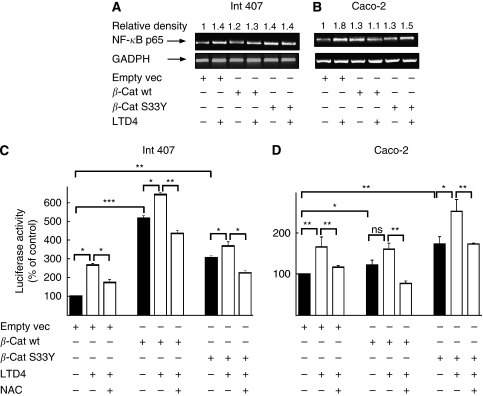
Activation of the p65 subunit of NF-*κ*B and the NF-*κ*B response element in cells overexpressing *β*-catenin. Int 407 (**A**, **C**) and Caco-2 (**B**, **D**) cells were transiently transfected with empty vector (empty vec), HA-wt-*β*-catenin (*β*-cat wt) or HA-S33Y-*β*-catenin (*β*-cat S33Y), and treated or not treated with 40 nM leukotriene D_4_ (LTD_4_) for 1 h. PCR was performed to estimate levels of p65 messenger RNA (mRNA) (**A**, **B**). The results shown are representative of three separate experiments. In (**C**) and (**D**) cells were transiently co-transfected with NF*κ*B-luc and Renilla, plus one of the following vectors: empty vector (empty vec), *β*-cat wt or *β*-cat S33Y. As indicated, the cells were the pre-treated or not with NAC for 15 min before incubation in the absence or presence of 40 nM LTD_4_ for 1 h. The luciferase values were measured and normalised to the *Renilla* values. The present data are given as means±s.e. of three separate experiments. ^*^P<0.05; ^**^P<0.01; ^***^P<0.005.

Oxidative phosphorylation is the main source of ROS production. Increased mitochondrial activity is expected to induce ROS production. As we observed earlier that LTD_4_ stimulation leads to an accumulation of *β*-catenin in mitochondria, we next examined whether LTD_4_ stimulation and *β*-catenin overexpression resulted in elevated levels of ROS. The mean fluorescence intensity value per cells for unstimulated empty vector was 11.2 relative units (RU), calculated by the image program ImageJ (W Burger & MJ Burge, NIH, USA). Extracellular addition of H_2_O_2_ was used as a positive control (mean fluorescent intensity per cell was 26.5 RU). Leukotriene D_4_ increased ROS production in Int 407 cells, although the effect was less prominent than in the positive control (mean fluorescence per cell 16.1 RU; Figure 4). Overexpression of *β*-catenin constructs also caused an increase in ROS production. The downstream target for ROS in many cell types is the NF-*κ*B family of transcription factors (Bubici *et al*, 2006). We found that overexpression of *β*-catenin resulted in the activation of the p65 subunit of NF-*κ*B (Figures 5A and B). Furthermore, the p65 subunit mRNA of NF-*κ*B is increased in LTD_4_-stimulated cells. A similar increase was observed after overexpression of the *β*-cat wt or *β*-cat S33Y constructs. Leukotriene D_4_ stimulation of cells overexpressing *β*-cat wt or *β*-cat S33Y construct did not significantly change the level of the p65 mRNA (Figures 5A and B).

**Figure 6:**
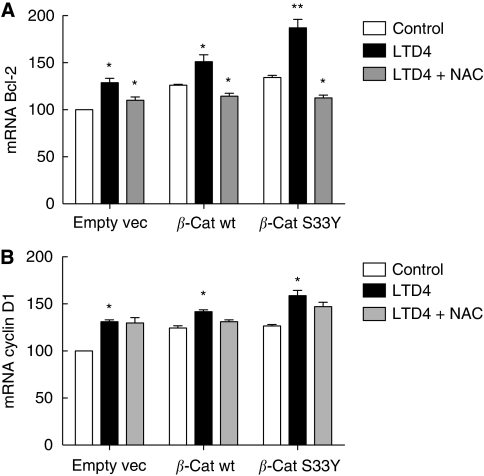
Effects of leukotriene D_4_ (LTD_4_) stimulation and *β*-catenin overexpression on Bcl-2 and Cyclin D1 expression. Caco-2 cells were transiently transfected with empty vector (empty vec), HA-wt-*β*-catenin (*β*-cat wt), or HA-S33Y-*β*-catenin (*β*-cat S33Y). As indicated, the cells were the pre-treated or not with NAC for 15 min before incubation in the absence or presence of 40 nM LTD_4_ for 18 h. Levels of Bcl-2 (**A**), and Cyclin D1 (**B**) messenger RNA (mRNA) in Caco-2 cells, were then measured by quantitative real-time PCR. The expression levels of the genes of interest were normalised to the expression of the reference gene *β*-actin and GAPDH. The presented data are given as means±s.e. of three separate experiments. ^*^P<0.05; ^**^P<0.01.

We next analysed whether LTD_4_ or *β*-catenin could affect the activity of NF-*κ*B. After LTD_4_ stimulation, we detected a three-fold increase in the activity of NF-*κ*B responsive elements in Int 407 cells and a 1.5-fold increase in Caco-2 cells (Figures 5C and D). Overexpression of *β*-cat wt or *β*-cat S33Y significantly increased activity of NF-*κ*B responsive elements in Int 407 (two-fold), and Caco-2 cells (1.4-fold). To analyse whether the increase in NF-*κ*B activity was ROS mediated, we used an antioxidant to block ROS formation. Preincubation with N-acetylcysteine (NAC) significantly reduced the effect of LTD_4_ and *β*-catenin overexpression in both cell lines (Figures 5C and D). We also analysed how NF-*κ*B activation affected its downstream target gene Bcl-2. Leukotriene D_4_ induced a significant increase in Bcl-2 expression that was further increased in cells transfected with either wt *β*-catenin or the S33Y−*β*-catenin mutant (Figures 6A). These effects of LTD_4_ were totally dependent on the LTD_4_-induced ROS production as shown by the use of the ROS inhibitor NAC that totally blocked the LTD_4_-induced increase in Bcl-2 expression (Figure 6A). To show the specificity of ROS/NF-*κ*B activation we also analysed the well-known target gene of *β*-catenin/TCF activity Cyclin D1. We found that both LTD_4_ and *β*-catenin overexpression significantly increased Cyclin D1 expression (Figure 6B). In contrast to the effects on Bcl-2 expression, the effects on Cyclin D1 expression were independent of ROS formation, as shown by preincubating the cells with the ROS inhibitor NAC (Figure 6B).

## Discussion

We have earlier observed that exposure of Int 407 cells to LTD_4_ causes translocation of *β*-catenin to the mitochondria, and association with the anti-apoptotic protein Bcl-2, thereby enhancing cell survival (Mezhybovska *et al*, 2006; Paruchuri *et al*, 2006). Here we suggest a possible mechanism linking *β*-catenin to survival signalling in the inflammatory environment. We observed an increase in complex I activity and an increase in ATP/ADP ratio in Int 407 and Caco-2 cells. It is worth mentioning that both Lef-1 and mitochondria transcription factor TFAM1 bears the similar HMG domain that is not only responsible for DNA binding but for protein–protein interaction as well. It has earlier been shown that ATP/ADP ratio is crucial for the decision of the cell's fate (Leist *et al*, 1997; Richter *et al*, 1996). Furthermore, Vrbacky *et al* (2003), showed that a decrease in ATP/ADP ratio in BSC-40 cells is a hallmark of apoptosis. It has been shown in tumour cells (HeLa, MCF-7, and HL-60) that mitochondrial ATP supported increased cellular proliferation rates (Guppy *et al*, 2002; Sweet and Singh, 1995). In agreement with earlier findings our data strongly indicate that an increase in ATP/ADP ratio favours survival signalling in the cell. Leukotriene D_4_ increased mitochondrial gene activity in both Int 407 and Caco-2 cells as the activity of the mtDNA encoding for complex I was also increased transiently in both Int 407 cells and Caco-2 cells.

Changes in mitochondria gene activity as well as mutations in the mitochondrial genome are reported for many cancers (Pelicano *et al*, 2004; Polyak *et al*, 1998; Waris and Ahsan, 2006). ND2 is overexpressed in human acute myeloid leukaemia cells (Matsunaga *et al*, 1996). An increased level of 16 s RNA was shown in polyps of familial polyposis coli patients (Yamamoto *et al*, 1989). Mutations in 16 s RNA has been reported for colorectal tumours as well (Polyak *et al*, 1998). *β*-Catenin overexpression in the non-transformed cell line, Int 407, had a dramatic effect on mitochondrial gene activity, but in cancer cells *β*-catenin overexpression did not show any significant differences. Caco-2 cells have higher levels of *β*-catenin compared with Int 407 cells and as a result, higher activity of the mitochondrial genome. Therefore, the effect of *β*-catenin overexpression on mitochondrial gene activity in Caco-2 cells was not as prominent as in Int 407 cells.

Reactive oxygen species are formed predominantly through oxidative phosphorylation. Formation of ROS is often increased in cancer in order to maintain cell growth and proliferation (Chen *et al*, 2007; Chen *et al*, 1998; Irani *et al*, 1997). Therefore, it was expected that ROS production would be elevated in this system. We observed increased ROS production in Int 407 cells (but not to the extent of the positive control, H_2_O_2_), upon LTD_4_ stimulation and after *β*-catenin overexpression. The cell fate decision for suicide or survival is dependent on signalling pathways activated upon oxidative stress. High doses lead to cell death while a moderate increase favours cell survival signalling (Martindale and Holbrook, 2002). The observed change in ROS was not as significant as in H_2_O_2_ stimulation, leading to the conclusion that a moderate increase in ROS levels in this system mediates survival signalling.

Earlier we showed that LTD_4_ facilitates survival signalling through both PI-3 kinase/Akt/GSK-3*β*- and PKC/Erk1/2-dependent mechanisms (Mezhybovska *et al*, 2006; Paruchuri *et al*, 2002). Erk activation as a result of oxidant injury was reported in 1996 (Guyton *et al*, 1996), resulting in cell survival and tumour growth. Akt activation in response to oxidant injury contributes to survival signalling in peroxide-induced apoptosis in a human glioblastoma cell line (Sonoda *et al*, 1999). Both Erk and Akt signalling has been shown to be upregulated upon LTD_4_ stimulation in the non-transformed intestinal cell line Int 407 (Mezhybovska *et al*, 2006; Paruchuri *et al*, 2002). In the Caco-2 cancer cell line, we observed ROS formation in untreated cells, in which the treatment with LTD_4_ did not have any additional effect (data not shown). This is not surprising since the elevated levels of ROS have been connected to cancer development for several decades (Pelicano *et al*, 2004). It was shown that extracellular addition of O_2_ resulted in activation of NF-*κ*B, and that this effect was ROS mediated (Cazals *et al*, 1999; Suzuki *et al*, 2000). We found an increase in activity of the p65 subunit of NF-*κ*B after LTD_4_ stimulation for 1 h in Int 407 and Caco-2 cells. Earlier reports from our group did not find any significant effect on NF-kB activation in Int 407 cells after 8 h or longer stimulation (Bengtsson *et al*, 2008). Overexpression of wild-type *β*-catenin- and *β*−catenin-S33Y constructs also activated the p65 subunit of NF-*κ*B. We also checked whether the activation of p65 subunit resulted in its increased activity. We used a luciferase assay to test activity of the p65 response element. The p65 responsive element activity was elevated after LTD_4_ stimulation as well as after *β*-catenin overexpression in both cell lines. There is still some confusion in the literature regarding possible cross-talk of NF-*κ*B and *β*-catenin signalling. *β*-Catenin has been reported to regulate NF-*κ*B in tumour cells (Amit and Ben-Neriah, 2003). Other reports suggest that NF-*κ*B is a regulator of the *β*-catenin pathway (Wang *et al*, 2007), whereas some studies reported that ROS activity is subject to negative feedback regulation by NF-*κ*B (Bubici *et al*, 2006). We used the antioxidant (NAC) to validate whether ROS is upstream or downstream of NF-*κ*B. p65 activation seems to be ROS mediated as the LTD_4_ mediated increase in ROS was reduced after antioxidant treatment. We also found that the well-known downstream target gene of NF-*κ*B activity Bcl-2 was induced by LTD_4_ stimulation or *β*-catenin overexpression. These effects were mediated through ROS production, which is in contrast to the effects of LTD_4_ stimulation and *β*-catenin overexpression on *β*-catenin/Tcf-mediated Cyclin D1 expression.

In conclusion, we have shown earlier that LTD_4_ induces *β*-catenin translocation to the mitochondria and increases survival by interacting with the cell survival protein Bcl-2. Here we describe for the first time that *β*-catenin is involved in signal transduction leading to increased activity of the respiratory chain, which in turn leads to an increased ROS production and as a consequence, NF-*κ*B activation. NF-*κ*B is one of the key players in inflammation, linking chronic inflammation and cancer development. Consequently, the present data add further support to the idea that NF-*κ*B activation might be crucial for LTD_4_-induced cell survival.

## Figures and Tables

**Table 1:** Overexpression of *β*-catenin increases mitochondria-encoded genes activity

**Gene**	**Int 407 (mean±s.e.)**	**Caco-2 (mean±s.e.)**	
*(A) Basal levels of genes expression*			
ND2	100±2.9	3200±9	
ND6	100±5.6	6000±9.2	
16 s	100±4	3300±5	
			
**Gene**	**Transfections**	**Unstimulated (mean±s.e.)**	**Stimulated with LTD_4_ (mean±s.e.)**
*(B) Int 407 cells*			
ND2	Empty vec	100	353±14.4^**^
	*β*-cat wt	605.6±20.7^***^	9937±207.2^***^; ^###^
	*β*-cat S33Y	804.1±47.6^***^	3448.2±123.8^***^; ^###^
ND6	Empty vec	100	377±5.6^***^
	*β*-cat wt	183.3±6.9^***^	19327.0±265.1^***^; ^###^
	*β*-cat S33Y	1163±37.0^***^	15693.6±120.9^***^; ^###^
16 s	Empty vec	100	163±4^**^
	*β*-cat wt	255.0±6^***^	6294.3±68^***^; ^###^
	*β*-cat S33Y	371.3±7.8^***^	2804.6±43.7^***^; ^###^
			
*(C) Caco-2 cells*			
ND2	Empty vec	100	163.3±8.4^**^
	*β*-cat wt	114.3±10.7	114.3±2.9^*^
	*β*-cat S33Y	170±20^**^	291.6±15.4^**^
ND6	Empty vec	100	135.6±7.7^**^
	*β*-cat wt	89.3±7.0	91.6±5.6
	*β*-cat S33Y	191.6±12.4^**^	191.4±11.4^**^
16 s	Empty vec	100	137±6.9^**^
	*β*-cat wt	148±9.2^**^	155.3±10^**^
	*β*-cat S33Y	199±12^**^	314±11^**^; ^###^

Levels of ND2, ND6, and 16 s mRNA in Int 407 and Caco-2 cells were measured after 5 days of cell growth, transfection was performed on the forth day in complete growth medium. Thereafter, cells were stimulated or not for 1 h with 40 nM LTD_4_. The expression of the genes of interest were normalised to the expression of the housekeeping gene actin B. The presented data are given as means±s.e. of four separate experiments. First, we compared *β*-catenin constructs transfections and stimulations with unstimulated empty vector control for each gene, ^*^P<0.05; ^**^P<0.01; ^***^P<0.005. Second, we compared stimulated with LTD_4_ to unstimulated for each vector transfection, ^###^*P*<0.005.
